# EBUS-TBNA在上腔静脉阻塞综合征诊断中的临床应用

**DOI:** 10.3779/j.issn.1009-3419.2013.09.08

**Published:** 2013-09-20

**Authors:** 胸怀 华, 玮 张, 惠民 冯, 秀峰 魏, 思杰 乔, 印 李

**Affiliations:** 1 450008 郑州，河南省肿瘤医院胸外科 Department of Toracic Surgery, Henan Cancer Hospital, Zhengzhou 450008, China; 2 450008 郑州，河南省肿瘤医院麻醉科 Department of Anesthesiology, Henan Cancer Hospital, Zhengzhou 450008, China; 3 450014 郑州，河南省军区医院病理科 Department of Pathology, Military Hospital of Henan Province, Zhengzhou 450014, China

**Keywords:** 气管腔内超声引导针吸活检术, 上腔静脉阻塞综合征, 临床应用, Endobronchial ultrasound-guided transbronchial needle aspiration, Superior vena cava obstruction syndrome, Clinical application

## Abstract

**背景与目的:**

纵隔淋巴瘤或右上肺癌是导致上腔静脉阻塞综合征（superior vena cava obstruction syndrome, SVCOS）的常见病因。气管腔内超声引导针吸活检术（endobronchial ultrasound-guided transbronchial needle aspiration, EBUS-TBNA）在纵隔占位疾病的诊断方面，与外科手术相当。本研究旨在探讨全麻状态下EBUS-TBNA在SVCOS临床病因诊断中的安全性及可行性。

**方法:**

2012年6月-2013年6月25例伴有SVCOS全麻状态下接受EBUS-TBNA的患者，其中男16例，女9例；年龄33岁-76岁，中位62.5岁。

**结果:**

24例病理学证实为恶性肿瘤，确诊率为96.0%（24/25），1例未能获得明确病理诊断；所有患者均未出现与操作相关的并发症，包括大量出血与气胸。

**结论:**

对伴有SVCOS的患者，全麻状态下EBUS-TBNA仍是一种确诊率高、安全可靠的微创检查方法，可作为其他手段不能明确病理诊断的常规检查。

## Abstract (en)

**Background and objective:**

Mediastinal lymphoma or right upper lung cancer is a common cause of superior vena cava obstruction syndrome (SVCOS). Endobronchial ultrasound-guided needle aspiration (EBUS-TBNA) biopsy is applied for the diagnosis of mediastinal mass disease. The aim of this study is to explore the safety and feasibility of EBUS-TBNA biopsy under general anesthesia for the clinical diagnosis of SVCOS.

**Methods:**

Twenty-five cases of SVCOS received EBUS-TBNA under general anesthesia between June 2012 and June 2013. The group consisted of 16 male and 9 female patients aged 33 years to 76 years, with a median age of 62.5.

**Results:**

Twenty-four cases were confirmed to be of malignant pathology, and one case failed to yield a clear pathological diagnosis. No patient experienced any complications related to the operation, such as heavy bleeding and pneumothorax.

**Conclusion:**

For patients presenting with SVCOS, EBUS-TBNA under general anesthesia is a safe and reliable inspection method of high diagnostic yield. This method can be used for routine examination when other means cannot obtain clear pathological diagnosis.

上腔静脉阻塞综合征（superior vena cava obstruction syndrome, SVCOS）是因上腔静脉或其周围病变引起上腔静脉不全或完全堵塞，导致经上腔静脉回流到右心房的血流不同程度受阻，从而表现为头颈、颜面及上肢淤血、水肿及上半身浅静脉曲张的一组临床综合征^[1]^。80%的上腔静脉阻塞源于纵隔淋巴瘤或右上肺癌侵犯纵隔，导致其管腔外压狭窄或管腔栓塞。通常情况下，未获得病理诊断前为了缓解上腔静脉梗阻的严重症状，多采用近距离照射进行治疗^[2]^。但是，组织学诊断对上腔静脉阻塞病因的鉴别优于经验性放疗，对部分肿瘤而言，尤其是小细胞肺癌（small cell lung cancer, SCLC）和非霍奇金淋巴瘤，系统性的化疗仍为其首选；并且，对纵隔病灶的照射往往会导致病理诊断的困难，使后续的治疗变得更为复杂^[3]^。气道肿瘤病理诊断可借助常规气管镜检查获得，但对伴有SVCOS的患者气管镜下活检被认作是高风险的操作，它可明显增加出血及气道水肿的风险^[4]^。随着气管腔内超声引导针吸活检术（endobronchial ultrasound-guided transbronchial needle aspiration, EBUS-TBNA）的临床逐渐推广及其对纵隔占位诊断地位的确立，EBUS-TBNA作为一种相对成熟、安全的操作，应有切实的理论基础可指导其在SVCOS的临床应用。但喉罩辅助通气全麻状态下为SVCOS的患者实施EBUS-TBNA，麻醉医师担忧甚多。现结合我院对25例SVCOS患者喉罩辅助通气全麻状态下EBUS-TBNA的实施情况，分析其在SVCOS中应用的安全性及可行性。

## 1 资料和方法

### 1.1 一般资料

2012年6月-2013年6月在河南省肿瘤医院发现有SVCOS并接受EBUS-TBNA的25例患者，男16例，女9例，年龄33岁-76岁，中位年龄62.5岁。所有患者术前常规接受胸部增强计算机断层扫描（computer tomography, CT），无明显重要脏器功能障碍及手术禁忌，且签署超声内镜下诊疗知情同意书。根据胸部CT扫描行放射治疗而无临床症状的SVCOS患者不在其中；事先知道SVCOS的病因或因良性因素比如导管相关造成的上腔静脉栓塞而在CT上无异常纵隔病灶的患者也应排除。

### 1.2 麻醉方法

静脉全麻辅助局部表面麻醉，监测患者心率、血压及血氧饱和度。为减少呼吸道及口腔内分泌物，术前静脉注射盐酸戊乙奎醚1 mg。依次静脉给予丙泊酚2 mg/kg-2.5 mg/kg、顺式阿曲库铵0.15 mg/kg-0.2 mg/kg及舒芬太尼2.5 μg/kg-4.0 μg/kg进行诱导。充分供氧2 min-4 min后置入喉罩，接Y型双腔支气管通气接头机控呼吸。术中以丙泊酚5 mg^-1^0 mg输注行麻醉维持，间断追加舒芬太尼、顺式阿曲库铵；同时以2%利多卡因2 mL-3 mL间断经支气管镜注入，减少患者呛咳、躁动。术毕视情况给予新斯的明及阿托品进行拮抗。

### 1.3 操作方法

常规先行普通支气管检查，对气道进行评估并对呼吸道内的分泌物进行充分吸引，以减少对后续操作的干扰。而后经口罩置入先端带有水囊、扫描频率为7.5 HZ的凸阵探头超声支气管镜（convex probe EBUS, CP-EBUS），以超声主机（EU-2000, Olympus, Tokyo, Japan）装置进行图像处理。实时监测状态下，经工作通道置入专用的22G细针进行穿刺，每个区域穿刺1次-5次。穿刺针的进针深度依超声下每区域淋巴结或肿块的大小而定，及时调整其穿刺角度和深度。但穿刺前进行多普勒扫描，以避免大血管损伤。抽吸标本常规行细胞学及病理学检查。部分标本进行免疫组化指标标记，以进一步明确诊断。本研究未进行及时快速细胞学评估（rapid on-site cytology evaluation, ROSE）。

### 1.4 统计学方法

采用描述性研究方法。诊断率以患者EBUS-TBNA确诊的百分比表示。

## 2 结果

25例伴有SVCOS的患者进行了25次EBUS-TBNA的规范性操作。右侧气管旁区域（#4R）靶病灶：CT扫描尺径2.55 cm-4.0 cm，平均3.15 cm；超声气道扫描的尺径0.89 cm^-2^.47 cm，平均2.17 cm。隆突下区域（#7）靶病灶：CT扫描尺径1.75 cm^-2^.80 cm，平均2.28 cm；超声气道扫描的尺径0.79 cm^-2^.02 cm，平均1.85 cm。4例仅行#4R区穿刺，1例仅行#7区穿刺，20例同时行#4R区及#7区穿刺。每个区域穿刺1次-5次，平均3次。24例获得明确诊断，其中SCLC 13例，肺腺癌2例，肺鳞癌5例，不典型类癌1例，弥漫大B细胞淋巴瘤1例，低分化非小细胞肺癌（non-small cell lung cancer, NSCLC）2例，明确诊断率96.0%（24/25）。1例未获得明确诊断。其中的1例患者实施EBUS-TBNA前在无病理诊断的前提下按淋巴瘤“CHOP”方案化疗1周期，而后免疫组化结果显示：SCLC(+)、CK(+)、EMA(+)、P63(散在少+)、TTF-1(+)、Syn(+)、CD56(+)和KI-67(70%+)，支持SCLC的诊断（图 1）。另1例因声音嘶哑伴胸闷的患者为迫切缓解症状，先给予经验行的放射治疗1周，EBUS-TBNA后的免疫组化结果为：Syn(+)、TTF-1(-)、NapsinA(+)、CD56(+)、CK7(-), 、P63(-)和CK19(-)，符合不典型类癌的诊断（图 2）。

**1:**
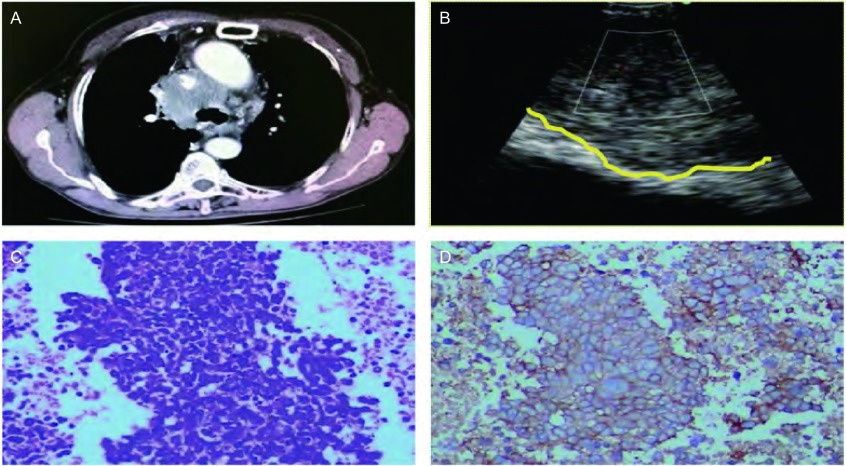
小细胞肺癌患者的影像、超声及病理资料。A：CT示纵隔内病灶致上腔静脉严重挤压；B：超声探测瘤体边界，多普勒显示瘤体血供；C：弥漫生长的小细胞，浆少，核浓染，并出现大片坏死（HE，×400）；D：免疫组化显示：SCLC(+)（2/HRP ×200）。 The imaging, ultrasonic and pathological data of the patient with small cell lung cancer (SCLC). A: CT showed the severe crushing superior vena cava was caused by the mediastinal lesion; B: Detected the boundary of tumor by Ultrasound, and showed the blood supply of tumor by Doppler; C: The small cells with less pulp and nuclear stain growed diffusely, and large areas of necrosis emerged (HE, ×400); D: Immunohistochemistry showed: SCLC(+)(2/HRP ×200). CT: computer tomography.

**2:**
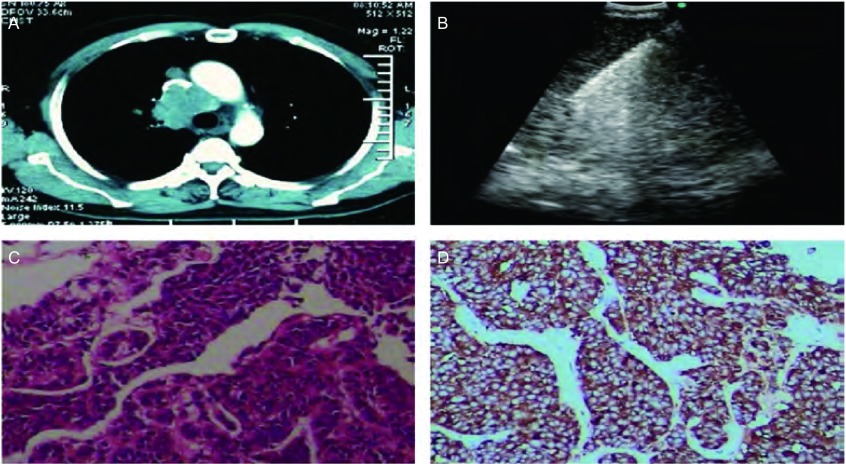
不典型类癌患者的影像、超声及病理资料。A：CT示纵隔淋巴结致上腔静脉近完全堵塞；B：穿刺过程中见到病灶内呈强回声影的细针；C：癌细胞排列呈不规则腺样、条索状，胞浆丰富，胞核大小较一致（HE，×400）；D：免疫组化显示：Syn(+)（2/HRP ×200）。 The imaging, ultrasonic and pathological data of the patient with atypical carcinoid. A: CT showed nearly completely blockage of the superior vena cava was caused by the mediastinal lymph nodes; B: The hyperechoic needle shadow in the lesion could be seen during puncture; C: Cancer cells with abundant cytoplasm arranged in irregular adenoid and cords, and the nucleus sizes were more consistent (HE, ×400); D: Immunohistochemistry showed: Syn(+) (2/HRP ×200).

没有发生与EBUS-TBNA操作本身相关的严重出血及死亡病例。3例患者出现血氧饱和度下降，积极增加供氧量后很快得以改善和维持（表 1）。

**1:** 25例实施EBUS-TBNA患者的临床资料 The clinical date of 25 patients undergoing EBUS-TBNA

Clinical features	Statistical results
Gender (M:F)	16:9
Age (Mean±SD)	60±13
Size of target lesion on CT scan	
Right paratracheal region, cm (Mean±SD)	3.15±1.18
Subcarinal region, cm (Mean±SD)	2.28±1.06
Diameter of target lesion under utrasound scan	
Right paratracheal region, cm (Mean±SD)	2.17±1.62
Subcarinal region, cm (Mmean±SD)	1.85±1.43
Number of patients with fine needle	
Only right paratracheal region puncture	4
Only subcarinal region puncture	1
Both right paratracheal and subcarinal regions puncture	20
Needle aspirated per station (median [range])	3 [1-5]
Final diagnosis (total 25)	
Small cell lung cancer	13
Lung adenocarcinoma	2
Squamous cell carcinoma	5
Atypical carcinoid	1
Diffuse large B-cell lymphoma	1
Non-small cell lung cancer, poorly differentiated	2
No diagnosis [*n* (%)]	1 (4.0%)
Complications	
Transient desaturation during procedure [*n* (%)]	3 (12.0%)
Severe bleeding or death	0
CT: computer tomography; EBUS-TBNA: endobronchial ultrasound-guided transbronchial needle aspiration	

## 3 讨论

上腔静脉位于右上纵隔，当右上肺肿瘤或其他部位肿瘤致腔静脉后淋巴结转移压迫上腔静脉，而侧支循环失代偿时则会出现面颈部充血水肿、结膜水肿、颈部增粗，并且因静脉受压还会引起血流淤滞、静脉压力增加，甚至血栓形成或出现脑水肿，导致患者迅速死亡^[5]^。SVCOS及时的诊断对选择适当的治疗是十分必要的，尤其是SCLC或淋巴瘤。30%的SCLC患者得到了系统性的治疗，即便是诊断为NSCLC的患者，通过追加的细胞类型也得到了适当的化疗。之前的放疗可能不是最佳的治疗方案，因为有近40%的情况是受照射的病灶影响了正确的病理分型^[6]^。Lee^[7]^在对恶性肿瘤接受EBUS-TBNA的一项研究中揭示了EBUS-TBNA前原发灶的部位和持续时间与最终的诊断无相关性。

至今支气管肺癌仍是导致SVCOS的最常见原因，常规的支气管镜活检似乎是接近这种情况的理想方法。然而，其诊断率不佳，波动于40%-67%，其原因可解释为管腔的外压而无支气管内的浸润。况且，活检后的出血可能会妨碍进一步对组织标本的提取而影响诊断结果^[8]^。在对SVCOS的研究中，虽然没有标准的治疗方案，但各种方法均可被采取作为一线研究^[9]^。从我们的经验看，大部分的患者最开始行EBUS-TBNA检查最终结果明确了诊断，队列的剩余部分作为未暴露组，他们是在常规支气管镜或胸腔穿刺术后进行EBUS-TBNA，其在SVCOS中总的确诊率为96.0%。如果有条件的话，ROSE也可同时进行，以确保获得足够的组织，并将针吸数量减少到最少。

在许多情况下，上腔静脉的外压是因来自纵隔、右侧气管旁或隆突下区域的病灶造成的，这些病灶通过常规的经支气管镜穿刺可轻易地获得，其诊断率达67.0%-96.0%^[10]^。与EBUS-TBNA相比，缺少EBUS监测的TBNA由于出血可造成操作提前的终止。另外，活检期间侧支循环的开放可能会增加大量出血的风险^[11]^。EBUS有内置的多普勒，它对接近或在病灶内的脉管系统实时鉴别，尤其是由于巨大纵隔肿块造成的解剖扭曲。在接受EBUS-TBNA检查的患者中未出现大出血，但活检时经常可碰到大出血这种并发症。

上腔静脉血管内支架作为SVCOS治疗的一种可行的治疗方案，可快速缓解症状，提高生活质量^[12]^。我们一位患者在行EBUS-TBNA前的6周已放过血管内支架，并接受低分子量肝素治疗，暂时性地阻止了病情进展，而后行EBUS-TBNA。但并无出血及血栓形成并发症。研究显示即便是有凝血性疾病存在，或有出血风险的脉管良性肿瘤，针吸也是安全的。有初步研究^[13]^认为在对纵隔肿瘤进行EBUS-TBNA之前放置血管腔内支架可能对非常严重的SVCOS患者来讲是种合理的方法。然而，我们的24例患者顺利的实施了EBUS-TBNA，事先并没放置上腔静脉内支架。事实上，虽然上腔静脉经皮支架置入有很高的成功率和极低死亡率，但譬如心包压塞之类的严重并发症可随时发生^[14]^。

尽管25例伴有SVCOS的患者全麻状态下顺利地接受了EBUS-TBNA，且未出现严重并发症，但仍需警惕急性肺栓塞、颅内高压等其他潜在风险的出现。需要注意的是操作过程中应严禁经上肢静脉输液给药，以免因回流不畅加重面颈部水肿，诱发颅内压增高而导致死亡。另外，对一般情况差、不能耐受平卧位的患者不应冒险尝试；术者应具备熟练的操作技能，将麻醉及操作时间控制在最短^[15]^。当然，单纯局麻镇静、镇痛状态下也可安全地进行EBUS-TBNA操作，清醒状态下可更好地有利于术后排痰，但患者对其耐受性及依从性可能不如全麻。总之，SVCOS患者缺乏如锁骨上淋巴结或胸膜积液等易接近的病灶部位时，全麻状态下EBUS-TBNA应是一种安全、微创、确诊率高的方法，并可获得足量的标本用于病理分型和分子分析。
